# Dimorphic Fungal Coinfection as a Cause of Chronic Diarrhea and Pancolitis

**DOI:** 10.1155/2011/960638

**Published:** 2011-08-07

**Authors:** Eduar A. Bravo, Arturo J. Zegarra, Alejandro Piscoya, José L. Pinto, Raúl E. de los Rios, Ricardo A. Prochazka, Jorge L. Huerta-Mercado, Jaime Cok, Martin Tagle

**Affiliations:** ^1^Department of Gastroenterology, Hospital Nacional Cayetano Heredia, Lima 31, Peru; ^2^Department of Pathology, Hospital Nacional Cayetano Heredia, Lima 31, Peru; ^3^Department of Gastroenterology, Clinica Anglo-Americana, Lima 18, Peru

## Abstract

*Histoplasma capsulatum* and *Paracoccidioides brasiliensis* are dimorphic fungi that cause systemic mycosis mostly in tropical South America and some areas of North America. Gastrointestinal involvement is not uncommon among these fungal diseases, but coinfection has not previously been reported. We report a patient with chronic diarrhea and pancolitis caused by paracoccidioidomycosis and histoplasmosis.

## 1. Introduction

*Histoplasma capsulatum* and *Paracoccidioides brasiliensis* are dimorphic fungi that cause systemic mycosis mostly in tropical South America and some areas of North America [1, 2]. These fungal agents share similar pathways to produce the infection through inhalation of the conidia of the mould into the alveoli, where the organisms change into the yeast form and then multiply through budding. Hematogenous dissemination occurs without clinical manifestations, and it can even develop many years later, depending on multiple factors related to the host's immune response (age, use of immunosuppressive drugs, concurrent diseases, and AIDS) [1, 3–5]. Gastrointestinal involvement is not uncommon among these fungal diseases [1, 6], but coinfection has not been previously reported.

## 2. Case Report

**Figure 1:**
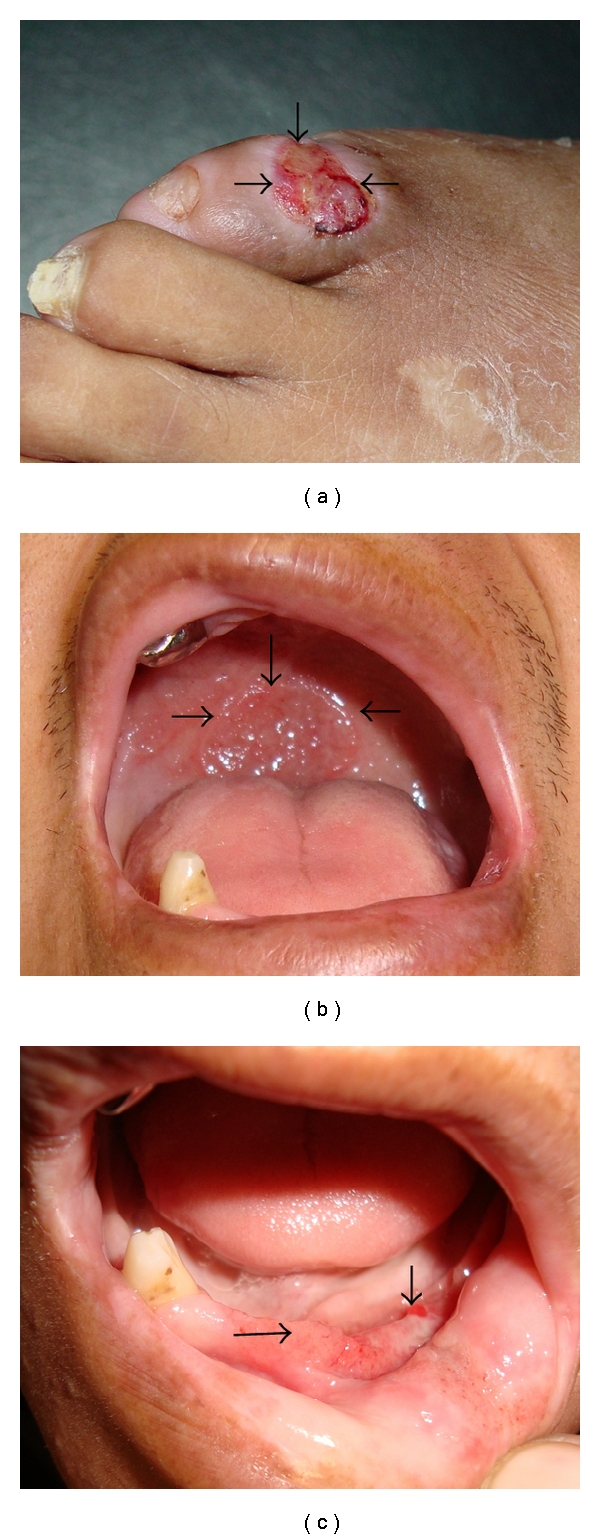
Clinical picture with arrows showing (a) chronic foot ulcer, (b) clear palate ulcer, and (c) mouth ulcer.

A 34-year-old male patient, from the state of Chanchamayo, Junin, Peru, with a medical history of chronic foot ulcer, was evaluated for one year with daily mucoid bloody diarrhea associated with intermittent infection by S*trongyloides stercoralis,* odynophagia, an ulcer in the upper palate and 12 kilogram weight loss. Over the last three months diarrhea episodes increased, and he presented with a nose ulcer. He denied chronic corticoid use, high-risk sexual behavior and had not been treated with immunosuppressive medications. Physical examination revealed a wasted patient with pale skin and upper palate, nose, mouth, and right foot ulcers (Figure 1). Blood testing showed anemia (hemoglobin 11 g/L) and hypoalbuminemia (albumin 16 g/L). He had more than 100 leucocytes and 30 red blood cells per field at the stool examination. The remaining of blood tests was normal. Parasitological studies were negatives. Chest X-ray was normal, and blood Elisa-HIV was negative. Qualitative assessment for HTLV-1 was positive. 

**Figure 2:**
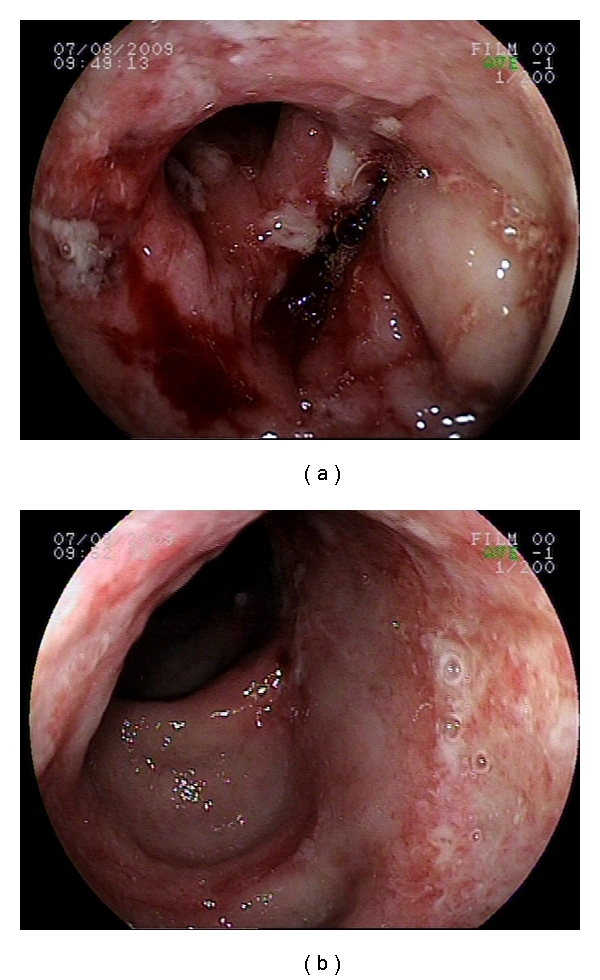
Colonoscopy showing (a) Intense inflammatory stenotic ulcer and (b) giant exudative colonic ulcer.

**Figure 3:**
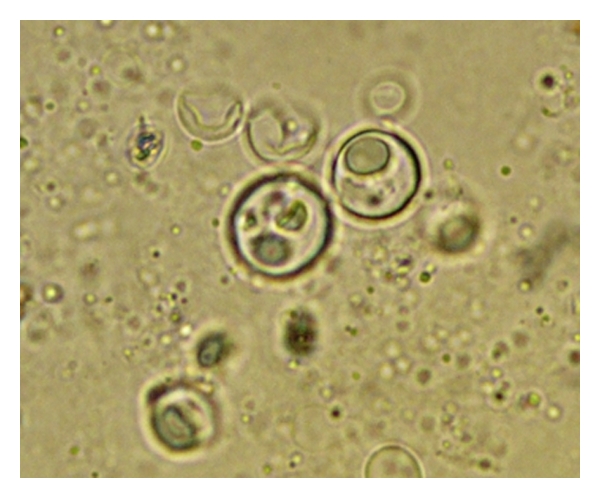
100x. Direct microscopic examination with typical double-wall* Paracoccidioides brasiliensis*.

Colonoscopy revealed multiple deep and large patchy exudative ulcers from rectum to cecum alternating with areas of normal mucosa (Figure 2). Direct microscopic examination revealed several double wall fungi with multiple gemmulation compatible with paracoccidioidomycosis (Figure 3). 

**Figure 4:**
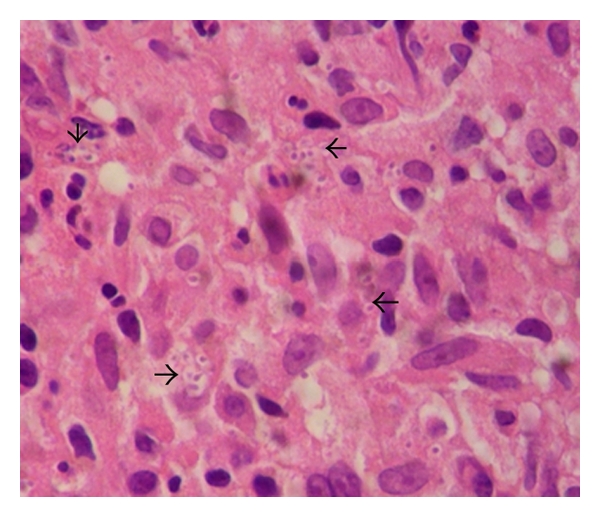
H&E 80x. Arrows showing intra- and extracellular small yeast of * Histoplasma capsulatum*.

**Figure 5:**
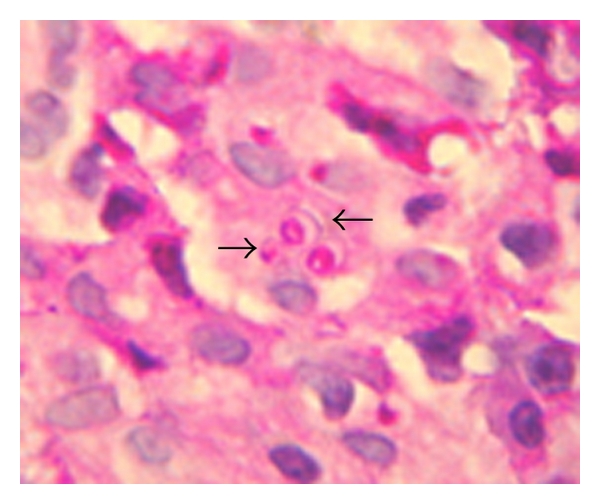
H&E 80x. Arrows showing intracellular *Paracoccidioides brasiliensis. *

Histopathology demonstrated acute infectious colitis with multiple granulomas rich in epithelioid cells and oval budding yeast cells consistent with *Histoplasma capsulatum* (Figure 4). In addition *Paracoccidioides* was found at a different site (Figure 5). Palate, nose, foot ulcer, sputum, and urine samples were positive for *Paracoccidioides*. Urine culture was positive for both *Histoplasma capsulatum* and *Paracoccidioides brasiliensis. *Blood cultures were negative for both fungi. The diagnosis of chronic progressive disseminated fungal coinfection was made. The patient received iv amphotericin B for 4 weeks as initial treatment with good response, and then he was discharged with oral itraconazole. He did not receive any treatment for HTLV-1 infections because he did not develop any mayor complications such as myelopathy/tropical spastic paraparesis (HAM/TSP) or adult T-cell leukemia/lymphoma (ATLL).

## 3. Discussion

Colonic fungal infection is not a common cause of colitis or chronic diarrhea; indeed colonic fungal coinfection has not been previously reported. Most patients with colonic infection due to *Histoplasma* or *Paracoccidioides* have developed multisystem disease [1, 4]. Moreover, even in immunocompromised patients, colonic involvement with histoplasmosis is rare [1, 7, 8]. Previous cases with colitis due to coinfections with histoplasmosis have been reported in immunocompromised patients (AIDS); Fan et al. [9] reported a 45-year-old man with colitis mimicking carcinoma caused by *Histoplasma* and cytomegalovirus, and Piscoya-Rivera et al. [10] reported a young man with lower gastrointestinal bleeding and coexistence of *Histoplasma* and *Mycobacterium tuberculosis.* In our case, the patient did not have any major immunocompromising conditions such as AIDS, chemotherapy, or immunosuppressive therapy, although we did not measure serum levels of immunoglobulin nor CD4/CD8 counts. However, he came from a well-known endemic zone of paracoccidioidomycosis which explained his condition as we previously reported for another patient [11]. There is not sufficient evidence to establish a relationship between these two fungi and HTLV-1 infection.

The symptoms most commonly described for GI histoplasmosis/paracoccidioidomycosis are nonspecific and include diarrhea, fever, abdominal pain, and weight loss [1, 6, 12]. Oropharyngeal involvement, found in 14–38% of individuals with disseminated disease, may offer a more readily accessible site for a diagnostic biopsy [13].

Diagnosis is suspected on clinical grounds, epidemiology, history, and imaging studies and confirmed with the identification of the fungus by culture, direct mycologic or histopathologic examination [1, 14]. Endoscopic appearance of colitis and colonic ulcers due to histoplasmosis/paracoccidioidomycosis is difficult to differentiate from tuberculosis, cytomegalovirus infection, inflammatory bowel disease, and even colon cancer [1, 3, 9], because they share similar features such as large, deep, and exudative ulcers with losing of vascular pattern. 

Both *Histoplasma* and *Paracoccidioides* are intracellular parasites, and they share similar patterns at histology such as granulomas rich in epithelioid and giant cells. An important clue to differentiate the two agents is that *Histoplasma* is a small capsulated yeast (2–4 *μ*m) and *Paracoccidioides* is a bigger yeast (10–40 *μ*m) with a double wall and gemmulation [1, 4, 11, 13].

The prognosis of disseminated histoplasmosis and paracoccidioidomycosis is poor, with a mortality rate of over 80% if untreated [1]. However, the use of amphotericin B can result in a clinical success rate of over 85% [15, 16]. In our case, the patient had a good clinical response with amphotericin B with clinical remission of loose stools and disappearance of oral ulcers. Although both histoplasmosis and paracoccidioidomycosis share similar treatment approaches, it is difficult to predict the outcome in patients with dimorphic fungal GI coinfection because of its rare occurrence.

##  Authors Contribution

Eduar Bravo, Arturo Zegarra, Alejandro Piscoya designed the research and wrote the paper. Ricardo Prochazka, Jorgue Huerta-Mercado and Jaime Cok analyzed the pathology data. Martin Tagle reviewed the paper.
